# Quality of Life, Active Ageing, Social Support and Loneliness in Older Adults Attending Day Centres: A Descriptive Analysis

**DOI:** 10.3390/healthcare14162559

**Published:** 2026-08-15

**Authors:** Julia Sánchez-Galloso, Andrés Arana-Rodríguez, José-Luis Sánchez-Ramos, Almudena Garrido-Fernández, Rocío Romero-Serrano, Francisca María García-Padilla

**Affiliations:** 1 Department of Nursing, School of Nursing, University of Huelva, Campus El Carmen, 21007 Huelva, Spain; 2 Centre for Research in Contemporary Thought and Innovation for Social Development (COIDESO), University of Huelva, 21007 Huelva, Spain

**Keywords:** quality of life, social support, loneliness, older adults, day centres, active ageing

## Abstract

**Highlights:**

**What are the main findings?**

- Older adults attending gerontological day centres reported moderate-to-high quality of life, with active ageing emerging as its strongest predictor.
- Greater perceived social support was associated with better quality of life, whereas higher social loneliness was associated with poorer quality of life.

**What are the implications of the main findings?**

- Interventions in gerontological day centres should prioritize strategies that promote active ageing, strengthen social support, and reduce social loneliness.
- These findings support the development of person-centred programmes aimed at improving quality of life among older adults attending day centres.

**Abstract:**

**Background/Objectives**: Population ageing has increased the importance of promoting wellbeing and healthy ageing. Gerontological day centres may support the wellbeing of older adults. This study aimed to examine quality of life, active ageing, perceived social support, and loneliness in older adults attending gerontological day centres and to identify the factors associated with quality of life. **Methods**: A cross-sectional descriptive study was conducted with 109 participants aged 60–98 years recruited from gerontological day centres in Huelva and Seville, Spain. Data were collected using the WHOQOL-BREF, the Multidimensional Scale of Perceived Social Support (MSPSS), the ESTE II Loneliness Scale, and the Active Ageing Scale. Descriptive and comparative analyses were performed, followed by multiple linear regression to identify factors associated with quality of life. **Results**: Mean scores were 14.46 (SD = 1.83) for quality of life, 3.06 (SD = 0.36) for active ageing, 10.14 (SD = 3.00) for social loneliness, and 5.62 (SD = 0.93) for perceived social support. The multiple linear regression model accounted for 30.8% of the variance in quality of life (adjusted R^2^ = 0.288), leaving approximately 69.2% of the variance unexplained by these factors. In this model, active ageing (β = 0.395; *p* < 0.001), social loneliness (β = −0.246; *p* = 0.004), and perceived social support (β = 0.188; *p* = 0.027) were independent predictors of quality of life. Bivariate analyses should be interpreted with caution as they were strictly exploratory. **Conclusions**: The study demonstrates that active ageing, low social loneliness, and solid perceived social support are the core multivariable predictors of quality of life among older adults in day centres. Secondary exploratory trends suggested minor baseline links with sleep duration, marital status, and employment status. Interventions should prioritize promoting active ageing and strengthening social networks, while future research needs to explore additional covariates to account for the remaining unexplained variance in well-being.

## 1. Introduction

The increase in longevity is one of the most significant demographic changes worldwide, with the population aged 60 years and over growing steadily and projections indicating a marked rise in the coming decades [1]. In southern Europe, this process has been more recent but exceptionally rapid, placing the region, alongside Japan, among the most rapidly ageing populations in the world. In Spain, the mean age has increased considerably in recent years, and both life expectancy and the proportion of older people are expected to continue rising, potentially reaching 30.5% by 2055 [2].

Population ageing is a global phenomenon associated with a growing demand for long-term care services. It is driven by multiple factors, including biological, familial, social and economic determinants, and leads to increasingly complex consequences at both the individual and societal levels [3]. In this context, health across the life course is now a central focus of research. Preventing chronic disease and improving the health of older populations represent key challenges for both scientific research and health and social care systems. Older adults require timely, comprehensive care that integrates biological, psychological and social dimensions [4]. The quality of professional practice and care provision is therefore critical, as this population tends to experience greater functional decline, higher levels of disability and more adverse events, both in hospital settings and in social and healthcare services such as residential facilities and Gerontological Day Centres (GDCs).

International evidence indicates that attendance at day centres for older adults has been associated with multiple benefits across psychosocial, cognitive and functional domains. The most consistently reported outcomes include improvements in perceived quality of life and functional capacity, which promote greater autonomy, enhance social perception, improve self-perception and subjective wellbeing, and contribute to reducing frailty. Improvements in overall functioning, general wellbeing and social integration among users have also been documented [5,6].

Given that quality of life is a multidimensional construct encompassing physical and psychological health, social relationships and engagement with the environment, its assessment should be considered an essential indicator of the effectiveness of geriatric services. In practice, however, systematic measurement of quality of life is not routinely undertaken in day centres for older adults [7,8]. In contrast to some prior literature, educational level did not significantly influence the observed associations in this sample, suggesting that the link between social loneliness and poorer quality of life remains stable regardless of the participants` educational background [9]. These findings highlight the importance of promoting social integration, interpersonal support and community participation as key components of active ageing, particularly in day centre settings where they may exert a meaningful influence on quality of life.

To the best of our knowledge, research simultaneously examining the relationships between social support, social loneliness and active ageing and their combined association with quality of life in older adults attending day centres remains limited, particularly within the Spanish context.

The present study therefore aims to describe these variables in older adults attending Gerontological Day Centres in the provinces of Huelva and Seville and to analyse their associations with relevant sociodemographic variables.

## 2. Materials and Methods

### 2.1. Design

This was a cross-sectional descriptive observational study.

### 2.2. Population and Sample

The study was conducted across seven Gerontological Day Centres (GDCs) located in the provinces of Huelva and Seville (Andalusia, Spain). The sample comprised 109 service users (52 from the province of Huelva and 57 from the province of Seville) who met the following inclusion criteria: regular attendance at a day centre and provision of written informed consent. The sole exclusion criterion was a diagnosis of moderate or severe cognitive impairment that could compromise comprehension of instructions or the capacity to provide valid informed consent. To ensure maximum clinical precision and comply with institutional confidentiality protocols, this screening was performed directly by the specialized medical and nursing staff of each participating gerontological day centre prior to data collection. The institutional clinical teams evaluated cognitive status through their official, updated records, utilizing standard validated tools in Spain (such as the Mini-Mental State Examination [MMSE] or the Pfeiffer Short Portable Mental Status Questionnaire) with established clinical cut-offs for moderate to severe impairment. Consequently, only users confirmed by the centers’ clinical staff to possess intact cognitive capacity or mild only status sufficient to reliably understand and respond to the self-report instruments were referred to the researcher. The screening and recruitment sequence followed a two-stage institutional workflow based on internal screening logs. Initially, the specialized staff identified a baseline pool of 125 potential participants across the selected institutions. During the first stage (preliminary clinical file screening), 11 users were excluded by the centers’ medical teams due to verified moderate-to-severe cognitive impairment, leaving a pool of 114 eligible candidates. In the second stage (formal approach by the researcher), 5 eligible individuals declined participation due to fatigue or lack of interest, while the remaining 109 eligible service users formally provided written informed consent and were fully analysed. Consequently, no potential participants who met the centers’ cognitive eligibility criteria and were officially referred to the researcher were excluded post-referral, yielding an authentic study response rate of 95.6% (109/114) among eligible candidates. Regarding the study period and participant flow, field research and data collection were conducted between November 2024 and January 2025. A non-probabilistic convenience sampling method was utilized to recruit participants based on institutional access and proximity. The seven Gerontological Day Centres (GDCs) were selected based on two specific criteria: (a) willingness of the center’s management to collaborate with the research project, and (b) presence of a stable, multi-disciplinary clinical team capable of pre-screening users. Across the selected institutions, the specialized staff identified a total of 125 potential participants who met the baseline criteria. Of these, 11 were excluded during the preliminary clinical record review due to moderate-to-severe cognitive impairment, and 5 declined to participate due to fatigue or lack of interest. Consequently, a total of 109 eligible service users were formally approached, provided informed consent, and were fully analysed, yielding a final response rate of 87.2% of the initially identified pool.

### 2.3. Variables and Data Collection

Data collection took place within the GDCs. Initial contact was made with each centre to obtain permission to approach eligible participants. Informed consent was subsequently obtained from those who met the inclusion criteria. Participants completed the questionnaire through individual interviews conducted by the same interviewer to minimise information bias. The study protocol was formally approved by the competent ethics committee. The study was formally approved by the Provincial Research Ethics Committee of Huelva (Reference: SICEIA-2025-002037), as detailed in the Institutional Review Board Statement section at the end of this manuscript.

Sociodemographic variables included age, sex, marital status, living arrangements, educational level, type and modality of the GDC attended, sleep duration (operationally categorized following the guidelines of the National Sleep Foundation into short sleep [≤5 h], recommended sleep [6 to 8 h], and long sleep [≥9 h]), length of attendance at the centre and employment status (categorized as domestic work exclusively versus employed or self-employed during active life) [10]. The primary study variables were also assessed: quality of life, perceived social support, social loneliness and active ageing. Quality of life was measured using the World Health Organization Quality of Life Brief Version (WHOQOL-BREF, which consists of 26 items across four domains, transformed into a 0 to 20 scale where higher scores indicate superior quality of life). Additionally, a global ‘Total Quality of Life’ index was operationally constructed for multivariate modeling purposes by calculating the direct mean arithmetic average across all 26 constituent items for each participant, achieving a high sample-specific internal consistency (α = 0.882) [11]; Perceived Social support using the Multidimensional Scale of Perceived Social Support (MSPSS, [12] comprising 12 items scored on a 1 to 7 mean scale where higher values reflect stronger support (sample-specific α = 0.908)); social loneliness using the Loneliness Scale for Older Adults (ESTE II, which includes 10 items where higher scores signify greater severity of social loneliness (α = 0.79) [13]); and active ageing using the Active Ageing Scale (EEA, featuring 37 items averaged into a 1 to 5 score range where higher marks denote a higher level of active ageing (sample-specific α = 0.776) [14]). All instruments have been validated in Spanish older populations and show adequate internal consistency and temporal stability. Although the WHOQOL-BREF is conventionally analyzed through its four independent domains, the utilization of a unified global Quality of Life index as a single dependent variable was justified to capture a comprehensive, holistic overview of the participants’ well-being and to maximize statistical parsimony within the multiple linear regression protocol, a composite approach extensively validated in recent gerontological and epidemiological literature [15].

### 2.4. Statistical Analysis

Descriptive analyses were performed, with frequencies and percentages reported for categorical variables and means and standard deviations for continuous variables.

Normality of continuous variables was assessed using the Kolmogorov–Smirnov and Shapiro–Wilk tests; the latter was used for subgroup analyses due to categories with fewer than 50 observations. This was supplemented by visual inspection of normal Q-Q probability plots, which showed no systematic departures from normality.

For variables meeting the assumptions of normality and homogeneity of variances (assessed using Levene’s test), parametric tests were applied: Student’s *t*-test for comparisons between two independent groups and one-way ANOVA for variables with more than two categories, with Bonferroni correction applied for post-hoc multiple comparisons.

Given the high number of multiple comparisons, all bivariate analyses performed (ANOVAs and *t*-tests) were considered strictly exploratory. Consequently, no alpha-level corrections were applied across the entire family of tests, and the findings are intended for hypothesis generation rather than causal inference.

Where normality was not met, non-parametric tests were used: the Mann–Whitney U test for dichotomous variables and the Kruskal–Wallis test for comparisons involving three or more groups. Regarding sample size validation, a post-hoc statistical power analysis was conducted utilizing G*Power, version 3.1.9.7 (Heinrich Heine University Düsseldorf, Düsseldorf, Germany) for the multiple linear regression model with three predictors. With a final sample of (*n* = 109) and an alpha level of 0.05, the study achieved a standard statistical power of 80% (1 − β = 0.80) power to identify a minimum detectable effect size of approximately Cohen’s (f^2^ = 0.103), which corresponds to a small-to-medium effect under standard assumptions.

A multiple linear regression analysis was subsequently performed, with quality of life as the dependent variable and perceived social support, social loneliness and active ageing as predictor variables. Regression assumptions were verified and multicollinearity assessed using the Variance Inflation Factor (VIF). Results were interpreted based on the coefficient of determination (R^2^), the unstandardised coefficient (B), the standardised coefficient (β) and a significance threshold of *p* < 0.05, with a 95% confidence level. All analyses were performed using IBM SPSS Statistics, version 30.0, (IBM Corp., Armonk, NY, USA). 

To ensure the psychometric validity of the findings, an internal sensitivity analysis was conducted by testing the models against the four official independent domains of the WHOQOL-BREF, confirming that the predictive patterns remained entirely stable across all sub-scales.

Sociodemographic variables (such as age, sex, marital status, and sleep duration), despite showing scattered associations in preliminary exploratory bivariate analyses, were intentionally excluded from this final multiple linear regression model. This decision was guided by statistical parsimony and sample size considerations (*n* = 109), ensuring the model maintained adequate statistical power and degrees of freedom to rigorously evaluate the core psychosocial predictors of interest without overparameterization.

Regarding missing data management, due to the face-to-face nature of the structured interview protocol executed individually by a single dedicated interviewer within the day centres, missing data were entirely absent from the final primary dataset, thereby eliminating the need for any statistical imputation procedures.

## 3. Results

### Characteristics of the Study Sample

A total of 109 service users attending Gerontological Day Centres in Huelva and Seville were included. As shown in Table 1, the sample comprised 63.3% women and 36.7% men, with a mean age of 81.08 years (SD = 7.05). The majority attended urban centres (77.1%), with the general modality predominating (88.1%) over the AFA modality (Alzheimer’s Family Association) (11.9%). A large proportion lived with a son or daughter (35.8%) or alone, and more than half were widowed (63.3%). Most participants had no formal education (60.6%), and regarding employment status during active life, 36.7% (*n* = 40) had engaged exclusively in domestic work, while 63.3% (*n* = 69) had been employed or self-employed. Although some educational sub-categories presented small sub-sample sizes (such as secondary education and non-formal education, both with *n* = 2), these brackets were intentionally kept disaggregated to guarantee complete reporting transparency and capture the authentic baseline heterogeneity of this older adult cohort. The mean length of attendance at the centres was 2.33 years (SD = 1.71), with one to two years being the most common. The results of the secondary bivariate analyses are presented below within an exploratory framework.

The overall mean quality of life score was 14.46 (SD = 1.83), with the environment domain showing the highest scores. The active ageing scale showed an overall mean scored of 3.06 (SD = 0.36), with an observed range spanning from 1.9 to 3.7. This global score was computed as an unweighted, direct arithmetic mean across all 37 individual items for each participant, rather than an average of aggregated dimension scores, ensuring a precise item-level distribution. Within its dimensions, higher values were observed in health and wellbeing (mean = 3.55, SD = 0.43) and lower scores in autonomy (mean = 2.61, SD = 0.29). Mean scores for social loneliness and perceived social support were 10.14 (SD = 3.00) and 5.62 (SD = 0.93), respectively.

Regarding associations between sociodemographic variables and quality of life, only one statistically significant difference was observed: total quality of life was associated with sleep duration (*p* = 0.021). Specifically, participants who slept longer reported higher total quality of life (mean = 15.46, SD = 1.92) than those who slept less (mean = 13.72, SD = 1.84) (see Supplementary Table S1 in Supplementary Material).

Further analysis of quality of life domains in relation to sociodemographic variables revealed significant differences in the psychological domain by age (*p* < 0.05). Psychological quality of life increased progressively with age and was significantly lower in the 60–70 age group compared with the 81–90 and 91–98 groups (*p* = 0.011 and *p* = 0.036, respectively), with the highest mean observed in the oldest age group (15.35 vs. 12.38 in the 60–70 group). In the environment domain, men had significantly higher scores than women (17.79 vs. 16.96; *p* = 0.030). In the social relationships domain, significant differences were observed by marital status (*p* = 0.013), with single participants scoring the highest absolute values (mean = 14.94, SD = 2.26), followed by married participants (mean = 14.21, SD = 2.72), who both scored higher than widowed participants (mean = 12.43, SD = 2.17). Differences were also observed by sleep duration (*p* < 0.001), with those who reported long sleep duration showing the highest scores (mean = 14.67, SD = 2.53) compared to those with short sleep duration (mean = 11.36, SD = 2.07), matching the full three-category breakdown detailed in Supplementary Table S2.

In the Social Participation dimension of active ageing, participants who had worked exclusively in the home (mean = 2.93) had similar scores to those who had been employed or self-employed (mean = 3.07). A similar pattern was observed in the Social Support dimension (mean = 2.80 vs. mean = 3.02). However, statistically significant differences were identified in both dimensions (*p* = 0.041 and *p* = 0.022, respectively), indicating that occupational history was associated with social participation and social support within the active ageing construct. Significant differences were also found in the Autonomy dimension, where participants who had worked exclusively in the home (mean = 2.51) showed different values to those who had been employed or self-employed (mean = 2.66), although the difference was significant (*p* = 0.007), suggesting that occupational history is also associated with autonomy within active ageing (see Supplementary Table S4 in Supplementary Material). Although employment status in this specific age cohort is closely linked with biological sex due to the historical context of Spain, these exploratory findings suggest a distinct psychosocial pattern regarding occupational backgrounds that warrants further investigation in adjusted future models. Additionally, although a baseline association was flagged between marital status and social participation (*p* = 0.043, see Table 2), subsequent pairwise post-hoc comparisons using Bonferroni corrections revealed no statistically significant differences between specific individual groups, indicating a weak overall trend rather than robust localized variances.

When total active ageing was considered, no significant differences were observed by sociodemographic variables (see Supplementary Table S3 in Supplementary Material).

Finally, differences in total social loneliness were observed according to marital status (*p* = 0.016), indicating that divorced participants reported the highest absolute social loneliness scores (mean = 11.60), followed by widowed participants (mean = 10.75), with widowed participants having higher scores than married participants (*p* = 0.009 for the post-hoc pairwise contrast) (see Supplementary Table S5 in Supplementary Material).

No significant differences in perceived social support were observed by sociodemographic variables (see Supplementary Table S6 in Supplementary Material).

Most sociodemographic variables, including educational level, living arrangements and day centre type (rural vs. urban), were not significantly associated with quality of life or active ageing. However, some variables were significantly associated with specific outcomes: sleep duration was associated with both total quality of life and social relationships; and employment status was associated with multiple dimensions of active ageing (see Table 2).

Simple regression analyses (Table 3) indicated that active ageing, social loneliness and perceived social support were significant individual predictors of quality of life. The multiple linear regression model (Table 4) explained a significant proportion of the variance in quality of life (** *R*^2^ = 0.308, Adjusted *R*^2^ = 0.288 **). Prior to interpreting the model, linear regression diagnostics were verified to ensure data robustness. Multicollinearity was formally ruled out, as the Variance Inflation Factors (VIF) for all predictors were strictly below 1.5. Furthermore, visual analysis of the standardized residual plots confirmed a satisfactory distribution of errors regarding normality and homoscedasticity, while data screening indicated no extreme cases or outliers distorting the stability of the regression coefficients. In this model, active ageing was the strongest factor associated, followed by social loneliness and perceived social support.

An internal domain-by-domain sensitivity verification confirmed that these multivariable associations were consistent across all standard sub-scales. Total Active Ageing (TOTALEA) maintained its position as the strongest independent predictor in all four individual domain models (*p* < 0.001 for Physical, Psychological, and Environment; *p* = 0.013 for Social Relationships), demonstrating that the main conclusions are robust and independent of the composite outcome score. Furthermore, to rule out demographic confounding, a sensitivity-adjusted regression model was executed by controlling for baseline age and sex (*F* (5, 103) = 10.209, *p* < 0.001). Neither sex (beta = 0.032, *p* = 0.700) nor age (beta = 0.154, *p* = 0.060) emerged as significant independent covariates. Crucially, the predictive strength and significance of the core psychosocial variables remained entirely robust and unchanged, with active ageing maintaining its position as the leading determinant (beta = 0.391, *p* < 0.001), followed by the remaining indicators (TOTALSS: (beta = −0.243, *p* = 0.004); TOTALEMAS: (beta = 0.182, *p* = 0.031)), demonstrating the structural stability of the primary findings.

## 4. Discussion

Compared with the existing literature, there is a scarcity of studies that jointly examine active ageing, social loneliness, perceived social support and quality of life in older adults attending day centres in this context. The present study therefore adds to the available baseline associations in this area, particularly within the Spanish context.

The descriptive findings indicate a moderately favourable profile. Total quality of life was moderate to high, with the environment domain scoring highest and consistent with studies showing that participation in community-based activities enhances perceived wellbeing [9]. Total active ageing was also moderate, with higher scores in health and wellbeing and lower scores in autonomy. This pattern has been reported in cross-sectional studies on active ageing and wellbeing, in which social and physical activity are associated with better perceived health and greater participation [16]. Social loneliness was also moderate, in line with European research suggesting that loneliness is relatively prevalent among older adults and may persist even in the presence of community services or support networks [17]. By contrast, perceived social support was relatively high, consistent with observational studies reporting similar scores [18].

Furthermore, the robust link identified between longer sleep duration and superior quality of life deserves careful academic consideration regarding its underlying mechanisms. In older cohorts, adequate and extended sleep duration operates as a vital biological and psychological buffer. From a physiological standpoint, optimal sleep architecture enhances emotional regulation and cognitive maintenance while significantly reducing chronic age-related micro-inflammation. Psychologically, achieving sufficient rest mitigates daytime fatigue and apathy, directly enhancing the energetic capacity of older adults to participate in daily activities and maintain social interactions. Consequently, restorative sleep does not merely reflect physical rest; it serves as a foundational pillar that directly feeds into the social, psychological, and environmental dimensions of subjective well-being within day centre environments.

Regarding the associations between variables, our findings suggest that quality of life in older adults attending GDCs is associated with active ageing, perceived social support and social loneliness. Sociodemographic factors such as age, marital status and educational level may also co-vary within these associations, reinforcing the biopsychosocial framework proposed by the World Health Organization for understanding wellbeing in older age [19].

The positive association between active ageing and quality of life is consistent with findings from Findling et al. [5] and other studies [18,20,21] which highlight how social participation and physical or cognitive activity sustain subjective wellbeing in later life. These findings support the idea that regular activity fosters life satisfaction and a sense of purpose, particularly in day centre settings where group activities and workshops facilitate socialisation and provide stimulation that supports healthy ageing.

The negative association between social loneliness and quality of life is also consistent with previous literature [22], which links reduced social connectedness with poorer emotional wellbeing and mental health. These findings highlight the importance of subjective perceptions of isolation and the need for programmes that foster meaningful and supportive relationships to enhance social connection in older adults [23].

Perceived social support appears to be a key protective factor, in line with studies highlighting its role in buffering the challenges associated with ageing [24]. Within day centre settings, interaction with professionals and peers may strengthen perceptions of both emotional and practical support, contributing to a more stable and sustained sense of wellbeing [5].

With respect to sociodemographic variables, the association between older age and greater psychological wellbeing is consistent with Socioemotional Selectivity Theory [25], which posits a shift towards emotionally meaningful goals in later life. Concurrently, the impact of marital status on social relationships further corroborates existing evidence identifying widowhood and the absence of a partner as risk factors for subjective distress [25,26], highlighting the central role of interpersonal relationships in wellbeing.

The absence of significant associations for some variables is also consistent with previous findings [27,28]. In care settings, factors such as educational level or living conditions may have less explanatory power, as these environments provide structured support and activities that act as compensatory factors. Not all sociodemographic characteristics therefore are associated with quality of life or active ageing to the same extent, and their interpretation should be situated within a broader framework that incorporates psychosocial and contextual factors.

Regarding the multiple linear regression model, it is crucial to acknowledge that while active ageing, social loneliness, and perceived social support emerged as key factors, they accounted for 30.8% of the variance (Adjusted R^2^ = 0.288) in quality of life. This indicates that a substantial 69.2% of the variability remains unexplained by these psychosocial dimensions. In line with gerontological literature, this remaining variance is highly likely driven by critical clinical and economic determinants that were not included in the present model. Factors common to this geriatric population—such as objective physical health status, multi-morbidity profiles, exact household income levels, specific functional independence thresholds, and sub-clinical depressive symptoms—exert a documented and powerful impact on the well-being of older adults. Future research incorporating these unmeasured variables alongside psychosocial predictors is highly warranted to construct a more exhaustive and comprehensive explanatory framework for quality of life in day centre settings.

This study has several limitations. The cross-sectional design precludes causal inference between the variables examined. In addition, due to this cross-sectional nature, reverse causation cannot be entirely ruled out, meaning that superior baseline quality of life could also foster enhanced perceptions of social support. Additionally, a potential psychometric overlap exists between the items of the MSPSS and the social support dimension of the Active Ageing Scale, which may artificially inflate their association and should be assessed with distinct tools in future research. Furthermore, the use of convenience sampling introduces a potential selection bias, as those who agreed to participate may differ systematically from those who declined, for example in terms of social support, health status or motivation. Crucially, the relatively small and non-probabilistic nature of this sample restricts the direct generalizability of our findings to the broader older population. Older adults attending Gerontological Day Centres (GDCs) possess distinct psychosocial, physical, and logistical profiles; they benefit from structured institutional engagement and daily socialization that may significantly buffer loneliness compared to completely independent community-dwelling peers, yet they concurrently exhibit higher functional frailty or dependency than those who do not require such services, while differing substantially from institutionalized individuals receiving full-time residential care. Data collection through personal interviews may also have introduced information bias, particularly social desirability bias, potentially affecting the validity of subjective measures such as quality of life and perceived social support. The sample size may additionally limit the statistical power of the study. Finally, the specific geographical context of day centres in the provinces of Huelva and Seville restricts the generalisability of the findings; multicentre studies with larger samples and longitudinal designs are needed to confirm and extend these findings.

## 5. Conclusions

The sample showed a moderately favourable profile, characterised by moderate-to-high quality of life, moderate active ageing, moderate social loneliness and relatively high perceived social support. Higher quality of life was associated with greater active ageing and higher levels of perceived social support, whereas social loneliness was negatively associated, indicating that a greater sense of loneliness is linked to lower wellbeing. These findings suggest that quality of life in older adults is associated with multiple social and personal factors, including activity, interpersonal relationships, and the support received from the environment.

Future research should examine how these variables interact across different cultural and socioeconomic contexts, as well as evaluate the effectiveness of targeted active ageing programmes and interventions aimed at strengthening social support networks in promoting quality of life within day centre settings. Longitudinal studies examining changes in quality of life over time would also be valuable, as would comparisons with older adults who do not attend day centres, in order to better understand the specific contribution of day centre services.

## Figures and Tables

**Table 1:** Sociodemographic Characteristics of the Participants.

Variables	*n* (%)	Mean	SD	Range
Qualitative variables				
Sex				
Female	69 (63.3%)			
Male	40 (36.7%)			
Marital status				
Single	5 (4.6%)			
Divorced	5 (4.6%)			
Married	26 (23.9%)			
Widowed	69 (63.3%)			
Partnered	4 (3.7%)			
Living arrangements				
Living alone	34 (31.2%)			
Living with a partner	21 (19.3%)			
Living with a child	39 (35.8%)			
Living with a partner and child (ren)	5 (4.6%)			
Living with others	10 (9.2%)			
Educational level				
No formal education (literate)	66 (60.6%)			
Primary education	26 (23.9%)			
Secondary education	2 (1.8%)			
Vocational training	5 (4.6%)			
Higher education (college or university)	8 (7%)			
Other non-formal education	2 (1.8%)			
Type of day centre				
Rural	25 (22.9%)			
Urban	84 (77.1%)			
Day centre modality				
AFA	13 (11.9%)			
General	96 (88.1%)			
Employment status				
Domestic work	40 (36.7%)			
Employed or self-employed	68 (63.3%)			
Sleep duration				
Short sleep (≤5 h)	17 (15.6%)			
Recommended sleep (6–8 h)	76 (69.7%)			
Long sleep (≥9 h)	16 (14.7%)			
Quantitative variables				
Age (years)		81.08	7.049	60–98
Length of attendance at the day centre (years)		2.33	1.71	0–9
QoL Physical health		12.95	2.7	6.8–20
QoL Psychological health		14.5	2.26	8–19.3
QoL Social relationships		13	2.54	8–20
QoL Environment		17.26	1.93	9.3–20
Total quality of life		14.46	1.83	9.9–19.3
AA Health and wellbeing		3.55	0.43	2–4.4
AA Social participation		3.02	0.34	1.8–4
AA Autonomy		2.61	0.29	1.5–3.4
AA Social support		2.95	0.5	1.8–4
Total AA		3.06	0.36	1.9–3.7
Total SL		10.14	3	5–18
Total MSPSS		5.62	0.93	2–7

Note. AFA: Alzheimer’s Family Association; SD: standard deviation; QoL: quality of life; AA: active ageing; SL: social loneliness; MSPSS: Multidimensional Scale of Perceived Social Support. Missing data were entirely absent across all descriptive sociodemographic variables (*n* = 109).

**Table 2:** Summary of statistical associations between sociodemographic variables and dependent variables and their dimensions.

Independent Variable	Dependent Variable	*F/t*	*η* ^2^	*p* Value
Age (years)	Psychological health (QoL)	*F =* 3.966	*η*^2^ = 0.102	0.010
Sex	Environment (QoL)	*t =* −2.195	*d =* −0.438	0.030
Marital Status	Social relationships (QoL)	*F* = 3.354	*η*^2^ = 0.11	0.013
	Social participation (AA)	*F =* 2.559	*η*^2^ = 0.09	0.043
	Social loneliness	*F* = 3.20	*η*^2^ = 0.11	0.016
Employment Status	Social participation (AA)	*t* = −2.068	*d* = −0.412	0.041
	Autonomy (AA)	*t* = −2.817	*d* = −0.561	0.007
	Social support (AA)	*t* = −2.320	*d* = −0.462	0.022
Sleep duration	Total quality of life	*F =* 4.015	*η*^2^ = 0.07	0.021
	Social relationships (QoL)	*F =* 7.911	*η*^2^ = 0.13	<0.001

Note. QoL = quality of life (WHOQOL-BREF); AA = active ageing; SL = social loneliness. Values correspond to statistical significance (*p* value) and effect size (*η*^2^) obtained from ANOVA F-tests.

**Table 3:** Simple linear regression results for predictors of quality of life.

Dependent Variable	Predictor Variable	Predictive Power (*R*^2^)	Unstandardized Coefficient (*B*)	Standardized Coefficient (β)	*p*-Value
Quality of life (QoL)	Active aging	0.192	2.23	0.438	<0.001
	Social loneliness	0.105	−0.198	−0.325	<0.001
	Perceived social support	0.084	0.568	0.290	0.002

Note. *R*^2^ = coefficient of determination; *B* = unstandardised coefficient; β = standardised coefficient; *p* = significance value.

**Table 4:** Multiple linear regression results for quality of life.

Dependent Variable	Predictor	*B*	SE	95% CI [Lower, Upper]	β	*p* Value	*R* ^2^	Adjusted *R*^2^
Quality of life	Total active ageing	1.998	0.414	[1.152, 2.796]	0.395	<0.001	0.308	0.288
	Total social loneliness	−0.150	0.051	[−0.249, −0.048]	−0.246	0.004		
	Total perceived social support	0.369	0.164	[0.033, 0.681]	0.188	0.027		

Note. *B* = unstandardised coefficient; SE = standard error; β = standardised coefficient; *p* = significance value; *R*^2^ = coefficient of determination; Adjusted *R*^2^ = adjusted coefficient of determination.

## Data Availability

The data presented in this study are not publicly available due to privacy and confidentiality considerations related to participant data. An anonymized version of the dataset is available from the corresponding author upon reasonable request.

## Supplementary Materials

The following supporting information can be downloaded at: https://www.mdpi.com/article/10.3390/healthcare14162559/s1, Table S1. Factors associated with total quality of life. Table S2. Quality of life dimensions (WHOQOL-BREF) by sociodemographic variables. Table S3. Factors associated with total active ageing. Table S4. Active ageing dimensions by sociodemographic variables. Table S5. Factors associated with total social loneliness. Table S6. Factors associated with total perceived social support.

## Abbreviations

The following abbreviations are used in this manuscript:

GDCs	Gerontological Day Centres
