# Supplementary material for: Quality of Life, Active Ageing, Social Support and Loneliness in Older Adults Attending Day Centres: A Descriptive Analysis
Source: Healthcare (Basel). 2026 Aug 15;14(16):2559. doi: 10.3390/healthcare14162559 (PMC13512316; doi:10.3390/healthcare14162559)
Supplement: Supplementary file 1 [file healthcare-14-02559-s001.zip › healthcare-4497529-supplementary.pdf]

Supplementary Material: Supplementary Tables

Supplementary Table S1. *Factors associated with total quality of life*

Variable	Category	*n*	Mean ($\bar{x}$)	SD	Range (min-max)	p value
**Age (years)**	**Total**		14.46	1.83	9.90–19.30	0.253
	60–70	2	13.48	1.62	11.10–16.00	
	71–80	14	14.36	1.80	11.30–17.70	
	81–90	29	14.60	1.79	9.90–19.30	
	91–98	33	15.15	2.29	12.00–18.80	
**Sex**	Women	69	14.30	1.84		0.227
	Men	40	14.74	1.79		
**Marital status**	**Total**		14.46	1.83	9.90–19.30	0.847
	Single	5	15.14	1.74	13.10–17.40	
	Divorced	5	14.06	3.01	11.60–19.30	
	Married	14	14.50	1.99	11.10–18.00	
	Widowed	15	14.39	1.75	9.90–18.80	
	Partnered	4	15.02	0.20	14.80–15.30	
**Living arrangements**	**Total**		14.46	1.83	9.90–19.30	0.389
	Living alone	32	14.51	1.87	9.90–19.30	
	Living with a partner	30	14.47	1.82	11.10–18.00	
	Living with a child	20	14.70	1.78	11.20–18.00	
	Living with a partner and child(ren)	13	14.30	2.83	11.30–18.80	
	Living with others	14	13.39	1.18	11.60–15.10	
**Educational level**	**Total**		14.46	1.83	9.90–19.30	0.654
	No formal education (literate)	66	14.63	1.77	11.20–18.80	
	Primary education	26	14.09	1.95	9.90–18.00	
	Secondary education	2	13.30	0.56	12.90–13.70	
	Vocational training	1	14.12	1.96	11.10–16.00	
	Higher education	2	14.90	2.16	12.80–19.30	
	Other non-formal education	12	13.80	1.13	13.00–14.60	
**Employment status**	Domestic work		14.71	1.91		0.274
	Employed or self-employed		14.30	1.79		

Variable	Category	*n*	Mean (x̄)	SD	Range (min-max)	p value
**Sleep duration**	Total		14.46	1.83	9.90–19.30	0.021
	Short/insufficient sleep	17	13.72	1.84	11.20–16.60	
	Recommended/optimal sleep	76	14.41	1.73	9.90–18.80	
	Long/prolonged sleep	16	15.46	1.92	12.00–19.30	
**Type of day centre**	Rural	14	14.62	1.80		0.623
	Urban	29	14.41	1.84		

Supplementary Table S2. *Quality of life dimensions (WHOQOL-BREF) by sociodemographic variables*

Dimensions	Category	*n*	Physical health Mean (SD)	Psychological health Mean (SD)	Social relationships Mean (SD)	Environment Mean (SD)
**Age (years)**	**Total**		12.94 (2.69)	14.50 (2.26)	13.00 (2.53)	17.26 (1.93)
	60-70	2	12.64 (2.44)	12.38 (2.44)	12.88 (1.49)	16.05 (1.79)
	71-80	14	12.62 (2.72)	14.29 (1.93)	12.96 (2.94)	17.61 (1.61)
	81-90	29	13.34 (2.87)	14.91 (2.33) [b]	12.93 (2.29)	17.26 (1.90)
	91-98	33	12.43 (2.69)	15.35 (1.88) [b]	13.81 (2.98)	16.78 (3.20)
	**Test statistic**		*F* = 0.700	*F* = 3.966	*H* = 0.932	*H* = 6.237
	p value		0.554	0.010	0.818	0.101
**Sex**						
	Female	69	12.66 (2.63)	14.40 (2.28)	12.96 (2.50)	16.96 (2.09)
	Male	40	13.45 (2.76)	14.67 (2.23)	13.08 (2.63)	17.79 (1.48)
	**Test statistic**		*t = -1.487*	*t = -0.613*	*t = -0.238*	*t = -2,195*
	p value		0.140	0.541	0.812	0.030

**Marital status**	**Total**		12.94 (2.69)	14.50 (2.26)	13.00 (2.53)	17.26 (1.93)
	Single	5	13.48 (2.45)	14.14 (1.93)	14.94 (3.04)	18.00 (1.32)
	Divorced	5	12.32 (4.72)	13.56 (2.36)	13.04 (4.07)	17.20 (1.64)
	Married	14	12.44 (2.46)	14.20 (2.56)	14.21 (2.72)	17.19 (1.76)
	Widowed	15	13.09 (2.71)	14.63 (2.22)	12.43 (2.17)	17.20 (2.11)
	Partnered	4	13.82 (1.08)	15.65 (0.70)	12.65 (1.30)	17.87 (0.47)
	**Test statistic**		*F* = 0.488	*F* = 0.671	*F* = 3.354	*F* = 0.302
	p value		0.744	0.613	0.013	0.876
**Living arrangements**	**Total**		12.94 (2.69)	14.50 (2.26)	13.00 (2.53)	17.26 (1.93)
	Living alone	32	13.70 (2.74)	14.67 (2.37)	12.73 (2.93)	16.96 (2.59)
	Living with a partner	30	12.75 (2.29)	14.31 (2.69)	13.71 (2.50)	17.11 (1.80)
	Living with a partner and child(ren)	20	13.01 (2.80)	15.01 (1.82)	13.01 (2.41)	16.90 (0.65)
	Living with others	13	10.40 (1.92)	13.46 (1.59)	13.08 (2.71)	16.50 (1.24)
	**Test statistic**		*F* = 2.422	*F* = 2.293	*F* = 0.643	*H* = 7.369
	p value		0.053	0.064	0.633	0.118

**Educational level**	**Total**		12.94 (2.69)	14.50 (2.26)	13.00 (2.53)	17.26 (1.93)
	No formal education (literate)	66	12.95 (2.82)	14.94 (1.95)	12.88 (2.44)	17.54 (2.26)
	Primary education	26	12.81 (2.26)	14.02 (2.58)	13.17 (2.43)	16.38 (2.26)
	Secondary education	2	12.85 (1.20)	13.00 (3.45)	11.35 (0.91)	16.00 (0.70)
	Vocational training	1	12.58 (2.82)	14.16 (2.32)	13.60 (2.90)	17.30 (0.97)
	Higher education	2	12.50 (2.12)	14.16 (2.32)	13.48 (3.94)	18.18 (1.53)
	Other non-formal education	12	12.60 (0.00)	12.35 (2.33)	13.35 (1.90)	16.75 (0.35)
	**Test statistic**		$F = 0.157$	$F = 1.787$	$F = 0.333$	$F = 2.002$
	p value		0.977	0.122	0.892	0.085
**Employment status**	Domestic work		12.81 (2.52)	14.92 (1.81)	13.46 (2.71)	17.28 (2.26)
	Employed or self-employed		13.02 (282)	14.29 (2.47)	12.69 (2.39)	17.25 (1.74)
	**Test statistic**		$t = -0.385$	$t = 1.359$	$t = 1.522$	$t = 0.084$
	p value		0.701	0.177	0.131	0.934

**Sleep duration**	**Total**		12.94 (2.69)	14.50 (2.26)	13.00 (2.53)	17.26 (1.93)
	Short/insufficient sleep	17	12.18 (3.56)	14.00 (2.24)	11.36 (2.07)	17.38 (1.87)
	Recommended/optimal sleep	76	12.83 (2.44)	14.46 (2.18)	13.02 (2.45)	17.14 (1.93)
	Long/prolonged sleep	16	14.31 (2.49)	15.21 (2.58)	14.67 (2.53)	17.68 (2.03)
	**Test statistic**		*F* = 2.898	*F* = 3.416	*F* = 7.911	*F* = 0.562
	p value		0.060	0.177	< 0.001	0.572
**Type of day centre**	Rural	14	13.13 (3.25)	14.76 (2.31)	12.85 (1.84)	12.85 (1.84)
	Urban	29	12.89 (2.52)	14.42 (2.25)	13.05 (2.71)	13.05 (2.71)
	**Test statistic**		*t = 0.396*	*t = 0.661*	*t = -0.351*	*t = 1.664*
	p value		0.693	0.510	0.726	0.099

Note. *Test statistics represent ANOVA (F-test), Student's t-test (t), Kruskal-Wallis (H), or Mann-Whitney (U) tests depending on data distribution and number of groups. Significant associations (p < 0.05) are highlighted.*

Supplementary Table S3. *Factors associated with total active ageing*

Variable		*n*	Mean ($\bar{x}$)	SD	Range (min-max)	p value
**Age (years)**	**Total**		3.06	0.36	1.90-4.60	0.486
	60-70	2	2.90	0.33	2.40-3.40	
	71-80	14	3.10	0.35	2.50-4.00	
	81-90	29	3.01	0.36	1.90-4.60	
	91-98	33	3.11	0.41	2.70-4.00	
**Sex**	Female	69	3.02	0.33		0.132
	Male	40	3.13	0.39		
**Marital status**	**Total**		3.06	0.36	1.90-4.60	0.974
	Single	5	3.10	0.40	2.50-3.60	
	Divorced	5	3.04	0.23	2.70-3.30	
	Married	14	3.03	0.35	2.40-4.00	
	Widowed	15	3.06	0.38	1.90-4.60	
	Partnered	4	3.15	0.17	3.00-3.30	
**Living arrangements**	**Total**		3.06	0.36	1.90-4.60	0.398
	Living alone	32	3.02	0.38	1.90-3.70	
	Living with a partner	30	2.98	0.26	2.40-3.40	
	Living with a child	20	3.12	0.34	2.60-3.40	
	Living with a partner and child(ren)	13	2.94	0.29	2.60-3.40	
	Living with others	14	3.16	0.54	2.90-3.60	
**Educational level**	**Total**		3.06	0.36	1.90-4.60	0.799
	No formal education (literate)	66	3.06	0.38	1.90-4.60	

	Primary education	26	3.01	0.31	2.50-3.50	
	Secondary education	2	3.00	0.70	2.50-3.60	
	Vocational training	1	3.18	0.46	3.00-3.50	
	Higher education	2	3.16	0.20		
	Other non-formal education	12	2.85	0.21	2.70-3.00	
**Employment status**	Domestic work		3.01	0.46		0.354
	Employed or self-employed		3.08	0.29		
**Sleep duration**	**Total**		3.06	0.36	1.90-4.60	0.507
	Short/insufficient sleep	17	3.02	0.42	1.90-3.60	
	Recommended/optimal sleep	76	3.04	0.36	2.40-4.60	
	Long/prolonged sleep	16	3.15	0.26	2.70-3.60	
**Type of day centre**	Rural	14	3.16	0.46		0.091
	Urban	29	3.02	0.32		

Supplementary Table S4. *Active ageing dimensions by sociodemographic variables*

Dimensions	Category	n	Health and wellbeing Mean (SD)	Social Participation Mean (SD)	Autonomy Mean (SD)	Social support Mean (SD)
**Age (years)**	**Total**		3.55 (0.43)	3.02 (0.34)	2.61 (0.28)	2.94 (0.50)
	60-70	2	3.23 (0.49)	2.84 (0.26)	2.52 (0.22)	2.95 (0.60)
	71-80	14	3.58 (0.40)	3.10 (0.37)	2.66 (0.30)	3.01 (0.52)

	81-90	29	3.57 (0.43)	3.01 (0.33)	2.58 (0.28)	2.92 (0.49)
	91-98	33	3.60 (0.38)	2.92 (0.19)	2.61 (0.28)	2.77 (0.29)
	**Test statistic**		*F* = 1.824	*F* = 1.798	*F* = 0.848	*F* = 0.600
	p value		0.147	0.152	0.471	0.616
**Sex**	Female	69	3.51 (0.45)	3.00 (0.35)	2.58 (0.30)	2.94 (0.49)
	Male	40	3.61 (0.37)	3.05 (0.32)	2.64 (0.24)	2.94 (0.51)
	**Test statistic**		*t = -1.083*	*t = - 0.781*	*t = -1.052*	*t = -0.001*
	p value		0.281	0.437	0.295	0.999
**Marital status**	**Total**		3.55 (0.40)	3.02 (0.34)	2.61 (0.28)	2.94 (0.50)
	Single	5	3.78 (0.40)	3.36 (0.51)	2.58 (0.40)	2.68 (0.58)
	Divorced	5	3.52 (0.31)	2.98 (0.24)	2.88 (0.19)	2.74 (0.53)
	Married	14	3.48 (0.44)	2.92 (0.27)	2.61 (0.27)	2.87 (0.43)
	Widowed	15	3.54 (0.44)	3.02 (0.34)	2.58 (0.28)	3.00 (0.40)
	Partnered	4	3.75 (0.10)	3.30 (0.29)	2.65 (0.19)	3.05 (0.40)
	**Test statistic**		*F* = 0.488	*F* = 2.559	*F* = 1.275	*F* = 0.961
	p value		0.744	0.043	0.284	0.432
**Living arrangements**	**Total**		3.55 (0.43)	3.02 (0.34)	2.61 (0.28)	2.94 (0.50)
	Living alone	32	3.47 (0.51)	3.01 (0.43)	2.55 (0.38)	3.07 (0.56)
	Living with a partner	30	3.46 (0.44)	2.98 (0.27)	2.60 (0.24)	2.85 (0.38)
	Living with a	20	3.65 (0.35)	3.07 (0.30)	2.67 (0.22)	2.94 (0.50)
	child		3.60 (0.40)	2.80 (0.24)	2.56 (0.16)	2.80 (0.48)
	Living with a	13				
	partner and					
	child(ren)		3.54 (0.37)	3.04 (0.26)	2.56 (0.18)	2.81 (0.50)

	Living with others	14				
	**Test statistic**		*F* = 1.011	*F* = 0.087	*H* = 3.231	*F* = 1.004
	p value		0.405	0.475	0.520	0.409
**Educational level**	**Total**		3.55 (0.43)	3.02 (0.34)	2.61 (0.28)	2.94 (0.50)
	No formal education (literate)	66	3.56 (0.40)	3.01 (0.29)	2.60 (0.28)	2.91 (0.47)
	Primary education	26	3.46 (0.08)	2.97 (0.32)	2.60 (0.28)	2.96 (0.52)
	Secondary education	2	3.60 (0.69)	3.25 (0.91)	2.35 (0.63)	2.80 (1.41)
	Vocational training	1	3.68 (0.40)	3.34 (0.68)	2.64 (0.24)	3.08 (0.37)
	Higher education	2	3.00 (1.41)	3.08 (0.27)	2.77 (0.28)	3.11 (0.59)
	Other non-formal education	12	3.15 (0.35)	2.85 (0.21)	2.40 (0.14)	2.95 (0.21)
	**Test statistic**		*F* = 0.801	*H* = 4.052	*F* = 1.100	*H* = 1.870
	p value		0.552	0.542	0.365	0.867
**Employment status**	Domestic work		3.44 (0.44)	2.93 (0.32)	2.51 (0.29)	2.80 (0.48)
	Employed or self-employed		3.60 (0.41)	3.07 (0.34)	2.66 (0.26)	3.02 (0.49)
	**Test statistic**		*t = -1.838*	*t = -2.068*	*t = -2.817*	*t = -2.320*
	p value		0.069	0.041	0.006	0.022

**Sleep duration**	**Total**		3.55 (0.43)	3.02 (0.34)	2.61 (0.28)	2.95 (0.50)
	Short/insufficient sleep	17	3.44 (0.60)	2.98 (0.42)	2.60 (0.38)	3.07 (0.56)
	Recommended/optimal sleep	76	3.54 (0.38)	3.00 (0.30)	2.59 (0.27)	2.88 (0.48)
	Long/prolonged sleep	16	3.66 (0.42)	3.17 (0.39)	2.67 (0.25)	3.10 (0.47)
	**Test statistic**		F = 1.097	*F* = 1.877	*F* = 0.483	*F* = 1.876
	p value		0.338	0.158	0.618	0.158
**Type of day centre**	Rural	14	3.53 (0.38)	3.00 (0.27)	2.61 (0.18)	2.93 (0.41)
	Urban	29	3.55 (0.44)	3.02 (0.35)	2.60 (0.31)	2.95 (0.57)
	**Test statistic**		*t = -0.190*	*t = -0.279*	*t = 0.117*	*t = -0.143*
	p value		0.849	0.781	0.907	0.887

****Note.*** Although the overall model was significant *(p = 0.043),* Bonferroni post-hoc comparisons did not reveal significant differences between specific pairs *(p > 0.05).*

Supplementary Table S5. *Factors associated with total social loneliness*

Variable		*n*	Mean ($\bar{x}$)	SD	Range (min-max)	p value
**Age (years)**	**Total**		10.14	3.00	5-18	0.928
	60-70	2	10.11	2.61	7-14	
	71-80	14	9.97	3.04	5-17	
	81-90	29	10.19	3.09	5-18	
	91-98	33	10.75	3.15	7-16	
**Sex**	Female	69	10.34	2.91		0.364
	Male	40	9.79	3.17		
**Marital status**	**Total**		10.14	3	5-18	0.029
	Single	5	9	2.64	5-12	
	Divorced	5	11.60	4.09	8-16	
	Married	14	8.76	2.26	5-13	
	Widowed	15	10.75	3.07	5-18	
	Partnered	4	8.25	1.25	7-10	
**Living arrangements**	**Total**		10.14	3.00	5-18	0.410
	Living alone		10.41	3.30	6-18	
	Living with a partner		9.14	1.93	6-13	
	Living with a child		10.28	2.90	5-16	
	Living with a partner and child(ren)		9.00	3.31	5-12	
	Living with others		11.40	3.83	6-16	
**Educational level**	**Total**		10.14	3	5-18	0.935
	No formal education	66	10.13	2.96	5-16	

	(literate)		9.84	2.86	5-15	
	Primary education	26				
	Secondary education	2	9.84	1.41	8-10	
	Vocational training	1	9.60	1.51	7-11	
	Higher education	2	11.75	4.62	7-18	
	Other non-formal	12	10.50	3.53	8-13	
	education					
**Employment status**	Domestic work		10.12	3.11		0.990
	Employed or self-		10.11	2.97		
	employed					
**Sleep duration**	**Total**		10.14	3	5-18	0.117
	Short/insufficient sleep	17	11.52	3.87	5-18	
	Recommended/optimal	76	9.86	2.87	5-16	
	sleep		10	2.30	5-14	
	Long/prolonged sleep	16				
**Type of day centre**	Rural	14	9.32	2.85		0.080
	Urban	29	10.39	3.02		

Supplementary Table S6. *Factors associated with total perceived social support*

Variable		*n*	Mean (x̄)	SD	Range (min-max)	p value
**Age (years)**	**Total**		5.62	0.93	2-7	0.292
	60-70	2	5.06	1.43	2-7	
	71-80	14	5.71	1	2-7	
	81-90	29	5.63	0.78	3.70-7	
	91-98	33	5.76	0.66	4.50-6.60	
**Sex**	Female	69	5.56	0.90		0.380
	Male	40	5.73	0.99		
**Marital status**	**Total**		5.62	0.93	2-7	0.188
	Single	5	5.05	1.26	3.70-6.40	
	Divorced	5	6.34	1.01	4.70-7	
	Married	14	5.80	0.74	4-7	
	Widowed	15	5.55	0.96	2-7	
	Partnered	4	5.62	0.53	5-6.20	
**Living arrangements**	**Total**		5.62	0.93	2-7	0.456
	Living alone	32	5.38	1.10	2-7	
	Living with a partner	30	5.74	0.65	4-7	
	Living with a child	20	5.73	0.90	2-7	
	Living with a partner and child(ren)	13	5.84	0.41	5.20-6.30 4-7	
	Living with others	14	5.67	1.09	2-7	
**Educational level**	**Total**		5.62	0.93	52-7	0.250
	No formal education	66	5.63	0.87	3.70-7	

	(literate)		5.65	0.84	5.70-6	
	Primary education	26	5.85	0.21	3.70-6.30	
	Secondary education	2	5.06	0.97	2-7	
	Vocational training	1	5.91	1.69	5-5.30	
	Higher education	2	5.15	0.21	2-7	
	Other non-formal education	12				
**Employment status**	Domestic work		5.69	0.81		0.547
	Employed or self-employed		5.58	1.00		
**Sleep duration**	**Total**		5.62	0.93	2-7	0.055
	Short/insufficient sleep	17	5.58	1.21	2-7	
	Recommended/optimal	76	5.55	0.84	2-7	
	sleep	16	6.01	0.97	3.70-7	
	Long/prolonged sleep					
**Type of day centre**	Rural	14	5.53	1.09		0.486
	Urban	29	5.65	0.88		
